# Genetic Parameters for Maternal Performance Traits in Commercially Farmed New Zealand Beef Cattle

**DOI:** 10.3390/ani11092509

**Published:** 2021-08-26

**Authors:** Franziska Weik, Rebecca E. Hickson, Stephen T. Morris, Dorian J. Garrick, Jason A. Archer

**Affiliations:** 1 School of Agriculture and Environment, Massey University, Private Bag 11222, Palmerston North 4442, New Zealand; R.Hickson@massey.ac.nz (R.E.H.); S.T.Morris@massey.ac.nz (S.T.M.); D.Garrick@massey.ac.nz (D.J.G.); 2 AbacusBio Ltd., P.O. Box 5585, Dunedin 9058, New Zealand; Jason.Archer@blnzgenetics.com

**Keywords:** beef cows, genetic parameters, correlations, reproduction, rebreeding, live weight, hip height, body condition score, maternal weaning weight

## Abstract

**Simple Summary:**

Enhancing maternal performance in a beef cattle enterprise can increase overall profitability. Knowledge of the degree of genetic variation in relevant traits is required to inform breeding decisions in commercial environments. The objective of this research was to examine the inheritance of maternal performance traits and to evaluate the trait complementarity among reproduction, live weight, hip height, body condition score and maternal weaning weight in 15-month-old heifers, 2-year-old cows and mature cows using data collected on commercial New Zealand hill country farms. Results from this study indicate that almost no genetic variation exists for pregnancy outcomes in 15-month-old heifers and mature cows under New Zealand farming conditions but there is potential to improve reproductive performance in 2-year-old cows through genetic selection. Cows with greater genetic potential for rebreeding performance after their first calving season were more likely to have greater live weight, hip height and body condition score as heifers but were unlikely to become larger cows at maturity. Cows with genetics for greater maternal weaning weight were more likely to carry lower body condition and those animals tended to show greater reproductive performance.

**Abstract:**

Maternal performance is a major driver of profitability in cow-calf beef cattle enterprises. The aim of this research was to evaluate the inheritance of maternal performance traits and examine the intercorrelation among reproduction, live weight, hip height, body condition and maternal contribution to calf weaning weight in 15-month-old heifers, 2-year-old cows and mature cows in New Zealand beef herds. Data were collected on a total of 14,241 cows and their progeny on five commercial New Zealand hill country farms. Heritabilities were low for reproductive traits in heifers and mature cows (0–0.06) but were greater in 2-year-old cows (0.12–0.21). Body condition scores were lowly (0.15–0.26) and live weights (0.42–0.48) and hip heights (0.47–0.65) highly heritable in heifers, 2-year-old cows and mature cows. Results indicate that 2-year-old cows with higher genetic potential for rebreeding ability may have greater genetic merit for live weight, hip height and body condition as heifers (r_g_ = 0.19–0.54) but are unlikely to be larger cows at maturity (r_g_ = −0.27–−0.10). The maternal genetic effect on weaning weight had a heritability of 0.20 and was negatively genetically correlated with body condition score in lactating cows (r_g_ = −0.55–−0.40) but positively genetically correlated with rebreeding performance (r_g_ = 0.48).

## 1. Introduction

Beef cows contribute indirectly to the profitability of a beef enterprise by producing calves that are either retained as herd replacements or finished for beef production. In New Zealand commercial farming operations, the profitability of beef cows is low due to low reproduction rates in terms of number of calves weaned per cow per year and high feed costs for raising herd replacements for the next generation. Thus, improving maternal and progeny performance is required to increase profitability of the cow-calf unit.

Potential antagonisms exist between increasing growth in finishing beef cattle systems and correlated response in live weight of mature cows [1]. The live weight of cows is often used as a predictor for feed requirements and represents the combined weight of muscle, fat, bones and internal organs [2] and varies by physiological state, frame size and gut-fill [3]. Heavier mature live weights are associated with higher energy requirements for maintenance, thus an increase would reflect higher costs for maintaining performance [4,5] and, therefore, may affect the profitability of the beef cow herd. In times of feed shortage, where feed requirements cannot be met, cows are required to mobilise body energy reserves. Body condition scoring is a measure of body fat reserves and is independent of the previously described factors influencing cow live weight [3,6,7,8]. Body condition is associated with reproductive performance of cows and a decline in energy reserves may adversely affect traits such as pregnancy rate or inter calving interval [9,10,11]. Dependent on breed and region, research has shown that an increase in live weight traits can, although only to a limited extent, adversely affect reproduction traits including days to calving (r_g_ = 0.07–0.08) [12,13] or pregnancy rate at 15 months of age (r_g_ = −0.32) [14] whereas other studies reported no impact on reproduction [15].

Selection for improved maternal performance is often inefficient as it relies on traits only measured on those females retained in the breeding herd and often expressed later in life [16]. Furthermore, most reproductive traits tend to be of low heritability [16,17,18], resulting in reduced prediction accuracy and, thus, constraining the rate of genetic gain. In commercial cattle environments, breeders are often concerned with the rebreeding ability of herd replacements after the first successful calving [19,20]. During that time, cows require energy levels to exceed maintenance requirements to achieve growth and milk production in combination with environmental challenges [21]. Without proper management, cows may in some years experience reduced conception rates in their second mating season [20].

Reports on the genetic variability of maternal performance in New Zealand beef cattle are relatively sparse and the inheritance of maternal performance traits has not yet been examined in commercial herds across a range of different environments and years. Better knowledge of the inheritance and relationships between key maternal performance traits in commercially farmed beef cattle is needed to make informed breeding decisions. Therefore, the objective of this study was to estimate genetic parameters for reproduction, live weight, hip height, body condition and maternal weaning weight traits measured in 15-month-old heifers, 2-year-old cows and mature cows in New Zealand beef herds.

## 2. Materials and Methods

### 2.1. Dataset and Animal Management

All measurements and related procedures were approved by the AgResearch Animal Ethics Committee (Approval numbers: 13358, 13373, 13394, 13693, 14031, 14311, 14588, 14851, 15153).

Data available for this research originated from an ongoing beef progeny test (BPT) by Beef + Lamb New Zealand Genetics conducted on five large-scale commercial hill country farms in New Zealand and initiated in 2014 [22]. The BPT was designed to compare the performance of cattle across a range of different breeds and environments.

Records were available from 2014 until 2021 for a total of 14,241 animals and the number of records is further outlined in Table 1. The data contained records on several traits for performance evaluation and records were obtained for the original population of cows in the project as well as all progeny resulting from each mating, and the corresponding pedigree was recorded. Calves were identified to the dam and sire by DNA parentage verification. Parentage of calves born prior to 2018 was determined by genotyping progeny and dams using a 120 SNP chip (Zoetis, Auckland, New Zealand) and sires through either 120, 10K or 50K SNP chips (Neogen, Gatton, Australia) and for calves born in 2018 or later was verified through 10K SNP chips for progeny and dams and 50K or above for sires (AgResearch GenomNZ, Dunedin, New Zealand).

All replacement heifers were naturally mated in their first two breeding seasons, at approximately 15 and 27 months of age. Only those cows that calved each year were retained in this study. From their third mating onwards, cows received a one-off artificial insemination (AI) at a synchronised oestrus at the start of mating followed by multi-sire natural mating for the remainder of the breeding season. Bull breeds used for AI were Angus, Hereford, Stabilizer, Charolais or Simmental and the foundation cows were Angus or Hereford. Details on the synchrony protocol are presented in Weik et al. [10]. The seasons aligned with extensive spring-calving production systems within the Southern Hemisphere such that the mating season began between November and January (dependent on the mating date policy on each individual farm). Pregnancy diagnosis (PD) was conducted approximately 90 days following AI and usually coincided with weaning of the calves. Trans-rectal ultrasound scans were conducted by an experienced commercial operator to confirm pregnancy and estimate fetal age. Cows diagnosed as not pregnant were culled following weaning of their previous calf. Culling was primarily conducted due to unsuccessful pregnancy but was also practiced because of health-related reasons. All cows calved in spring with the calving season ranging from September until November across all herds. Birth dates were not recorded but calculated for each calf based on the fetal age estimated at PD and assuming a 282 day gestation length [23] whereas only birth years were available for the original population of cows.

Animals were kept on pasture year round with little to no supplementary feed. No data have been collected on pasture availability and feed quality due to cattle grazing extensive hill country pastures.

### 2.2. Trait Definitions

The number of observations, range of data measures, means and standard deviations (SD) for recorded traits are outlined in Table 1.

Maternal traits were recorded for all cows present in the herd on the recording day. Traits were pregnancy rate of 15-month-old heifers (HP), days to conception in 15-month-old heifers (DtCH), rebreeding performance in 2-year-old cows (RB), days to conception in 2-year-old cows (DtC2), pregnancy rate of mature cows (PR), live weight of 15-month-old heifers (HWT), body condition score of 15-month-old heifers (HBCS), hip height of 15-month-old heifers (HH), live weight of 2-year-old cows (WT2), body condition score of 2-year-old cows (BCS2), hip height of 2-year-old cows (HH2), mature cow live weight (MWT), mature cow live weight adjusted for body condition score (MWT_BCS_), mature cow live weight adjusted for hip height (MWT_HH_), body condition score of mature cows (BCS), hip height of mature cows (MHH) and weaning weight of calves (WWT). Except for height records where data recording started in 2017 all other traits were recorded throughout the entire project. Recording dates for individual traits differed among farms involved in the BPT but were consistent for all cows within farm and year.

Observations for pregnancy outcomes (HP, RB, PR) were recorded as binary traits and were either 0 or 1 coded to represent unsuccessful and successful results, respectively. The HP relates to the percentage of naturally-mated heifers recorded as pregnant among all heifers present at PD conducted between 370 and 454 days of age. Likewise, RB describes the ability of a cow to successfully rebreed between 745 and 841 days of age and is defined as the percentage of all 2-year-old cows recorded as pregnant of all 2-year-olds present at PD. The trait PR was the percentage of cows aged 3 years or older present at PD that were diagnosed as pregnant.

The reproductive traits DtCH and DtC2 were defined as the number of days from the start of the mating season to the conception day in 15-month-old heifers and 2-year-old cows, respectively. Both measures were from calculations based on estimated fetal age recorded at PD used to ascertain probable conception dates. The date the first female within a mating contemporary group (CG) conceived was taken to be the start of the breeding season for that CG. Further information on CG assignment is provided in the data editing section. To allow for the inclusion of non-pregnant cows in the analyses, cows that failed to conceive were assigned a penalty of 21 days from the last conception date within their CG [13,24].

Live weights (HWT, WT2 and MWT) were recorded using electronic scales. The traits HWT and WT2 were defined as the live weight of females prior to their first or second mating season, respectively. The trait MWT was defined as the live weight of a cow from three years of age. Measurements for MWT were recorded at three timepoints throughout the annual production cycle: prior to mating (November–January), at weaning of the calf (February–April) and prior to calving (July–September) and were included as repeated measures in the analyses. Similarly, body condition score traits (HBCS, BCS2 and BCS) were recorded at the same timepoints within the production cycle and females were included according to their age as previously described for live weight records. Data for body condition scores were obtained by visual assessment based on a 1 to 10 scale (1 = emaciated and 10 = obese, Hickson et al. [8]). Scoring was conducted by an experienced scorer or by the farmer after training and under regular calibration to the trained scorer. Hip height records were obtained once a year around calving on a continuous scale using a tape measure. Based on the age of the cow, records were either HH, HH2 or MHH, according to the grouping criteria used for live weight and body condition score records. Records for MWT, BCS and MHH were adjusted to 5 years of age [25] prior to analysis by fitting a fixed effects model with age and CG as factors in the model. Further adjustments were applied for MWT to either a constant body condition score of 6 or hip height of 130 cm and they are referred to as MWT_BCS_ and MWT_HH_, respectively. The procedure used was the same as the 4-step procedure presented by Reverter et al. [26] using linear and quadratic effects for the covariate (BCS or MHH), with the modification that adjustments were obtained on an individual animal basis as opposed to a CG mean to account for within CG variation.

Weaning weight of calves was recorded at weaning, at which time the calves’ age varied from 110 to 228 days. Linear adjustments to a constant age of 200 days of age were applied using the same method previously described for MWT adjustments to a constant BCS or MHH following the approach by Reverter et al. [26]. The ancestry of animals born within the BPT was generally traced back only one generation, but progeny from naturally-mated heifers with own pedigree records were weighed at weaning and matched to their sire and dam to allow estimation of the maternal effect on calf weaning weight. The direct additive genetic effect of WWT is referred to as WWT_D_ and the maternal component of weaning weight as WWT_M_. The WWT_M_ describes the maternal contribution of the dam to the 200 day weight of the calf (descriptive of genetic potential for milk production [27,28]).

### 2.3. Data Editing

Recording errors were removed from the existing dataset prior to analyses and twin births were deleted.

For animals that were 2 years of age or younger, the estimated birth date as determined by fetal age scanning was used to derive age at data recording in days from birth. For mature cows, the exact birth date was not known and only the birth year was recorded. Consequently, the recorded birth year was used to compute age of animals in years for traits that were measured on mature cows (MWT, BCS, MHH and PR). Similarly, birth years were used to calculate age of dam. Grouping was only applied to age-in-years parameters such that each individual age represented a separate age class but animals older than 12 years of age were grouped together due to a limited number of animals in higher age classes.

**Figure 1:**
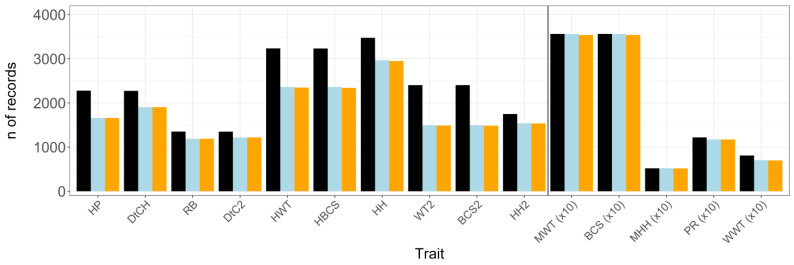
Original size of dataset (black bar) and number (n) of records after animals with missing contemporary group (CG) and/or age of animal or dam information (blue bar) as well as outliers (orange bar) have been removed from the dataset; n of records were divided by 10 for traits presented to the right of the divide; HP = pregnancy rate of 15-month-old heifers; DtCH = days to conception in 15-month-old heifers; RB = rebreeding performance in 2-year-old cows; DtC2 = days to conception in 2-year-old cows; HWT = live weight of 15-month-old heifers; HBCS = body condition score of 15-month-old heifers; HH = hip height of 15-month-old heifers; WT2 = live weight of 2-year-old cows; BCS2 = body condition score of 2-year-old cows; HH2 = hip height of 2-year-old cows; MWT = mature cow live weight; BCS = body condition score of mature cows; MHH = hip height of mature cows; PR = pregnancy rate of mature cows; WWT = weaning weight of calves.

Definitions for CGs are shown in Table 2. Animals with missing information required to define CG or CGs that contained only one animal were removed from the analyses. For binary traits, CGs that contained only the same values (0 or 1) for all animals were excluded. Possible outliers were excluded from the dataset by removing any observation further than three standard deviations from the CG mean [29]. The original number of records and the size of the dataset following data editing are shown in Figure 1.

All traits were examined for the presence of heterogeneous variances. Linear regression was used to evaluate the relationship between CG mean and SD and a significant relationship was considered evidence for deviation from homogeneity [30,31]. Traits were scaled to homogenize the variances where appropriate [29] based on the deviation of each record from the CG mean to the average of the entire dataset [32].

### 2.4. Statistical Analysis

Data editing and pre-adjustments of phenotypes were conducted using R version 3.6 [33]. (Co)variance components were estimated using various animal models in the ASREML 4.1 software package [34]. Heritability, repeatability and correlations (genetic and phenotypic) were calculated from the estimates with their approximate standard errors.

The models used for estimation of genetic parameters were of the general form:

(1) $${y=Xb+Z}_{a}u_{a}{+Z}_{m}u_{m}{+Z}_{\mathrm{pe}}u_{\mathrm{pe}}{+Z}_{\mathrm{me}}u_{\mathrm{me}}+\varepsilon$$

where y is the vector of pre-adjusted observations; X is an incidence matrix relating the fixed effects in b to the observations in y; $Z_{a}$, $Z_{m}$, $Z_{\mathrm{pe}}$ and $Z_{\mathrm{me}}$ are the incidence matrices relating the random effects $u_{a}$ for direct additive genetic, $u_{m}$ for maternal genetic, $u_{\mathrm{pe}}$ for permanent environmental and $u_{\mathrm{me}}$ for maternal environmental effects to observations in y; and ε is the vector of residual effects unique to each observation in y.

Expected values of y and variances for the random effects included in the model were assumed to be as follows:

(2) E[y]=Xb,

(3) $$\mathrm{var}\left[\begin{array}{c} a\\ \begin{array}{c} m\\ \mathrm{pe}\\ \mathrm{me}\\ \varepsilon \end{array} \end{array}\right]=\left[\begin{array}{ccccc} {A\sigma }_{a}^{2} & {A\sigma }_{\mathrm{am}} & 0 & 0 & 0\\ {A\sigma }_{\mathrm{am}} & {A\sigma }_{m}^{2} & 0 & 0 & 0\\ 0 & 0 & {I\sigma }_{\mathrm{pe}}^{2} & 0 & 0\\ 0 & 0 & 0 & {I\sigma }_{\mathrm{me}}^{2} & 0\\ 0 & 0 & 0 & 0 & {I\sigma }_{\varepsilon }^{2} \end{array}\right]$$

where A is the numerator relationship matrix, I are identity matrices with their order equal to the number of observations, $\sigma_{a}^{2}$ the additive genetic variance, $\sigma_{m}^{2}$ the maternal genetic variance, $\sigma_{\mathrm{am}}$ the direct-maternal genetic covariance, $\sigma_{\mathrm{pe}}^{2}$ the permanent environmental variance, $\sigma_{\mathrm{me}}^{2}$ the maternal environmental variance and $\sigma_{\varepsilon }^{2}$ the residual variance.

Given the amount of data and number of traits, a single multivariate analysis was not feasible such that a variety of uni- and bivariate animal models were used to allow computation of genetic parameter estimates. Variance components for heritability and repeatability estimates were obtained from univariate animal models including all traits on the observed scale. In a second approach all binary traits were analysed using threshold models with a logit-link function. Heritabilities for threshold traits were estimated on the underlying (logit) scale ($h_{L}^{2}$) as follows:

(4) $$h_{L}^{2}=\frac{\sigma_{a}^{2}}{\frac{\pi^{2}}{3}{+\sigma }_{a}^{2}}$$

where $\pi^{2}$/3 is the residual variance on the underlying scale [34,35].

The convergence criterion was the default value used by ASREML such that convergence was presumed when the log-likelihood changed less than 0.002 times the number of iterations and the change of individual variance parameter estimates was below 1%. Initial analysis of variance components considered bivariate animal models including one maternal trait plus WWT to account for any selection prior to data recording. However, these models failed to converge in some cases or estimates converged close to a boundary of the parameter space. For those models that did converge, variance components varied only slightly from the univariate models, such that further analyses were conducted using only single-trait models. Genetic and phenotypic correlations between trait pairs were obtained by estimating (co)variance components from bivariate analyses. Linear animal models were assumed among all traits [35]. The inclusion of reproductive traits in bivariate analyses was limited to those with heritabilities greater than 0.05 [14].

Fixed and random effects for each trait are displayed in Table 3. Genetic parameters were analysed on an across-breed basis and a breed percentage and heterosis were fitted for each trait. For those traits that were not pre-adjusted to a standard age, age in days was fitted as a covariate in the final model for traits measured on animals 2 years of age and younger and age in years was fitted as a factor for mature cow traits. Ancestors were traced back up to two generations. The A matrix comprised 14,241 individual animals including 423 sires and 4473 dams. No back pedigree was available for the original population of cows in the project, or the sires used for mating such that the base generation was assumed to be unrelated.

## 3. Results

### 3.1. Univariate Analyses

Estimates of variance components, heritabilities and repeatabilities evaluated in this study are presented in Table 4 for all traits.

Heritability estimates were low or zero for the binary traits HP (0.00), RB (0.14) and PR (0.00) on the observed scale. Estimates obtained on the underlying scale using a logit link function differed from those on the observed scale only marginally. Estimates were slightly higher for HP on the logit scale with 0.06 and similar for RB with 0.12. The approximate standard errors obtained on the underlying scale, however, were larger for both traits (0.08–0.11) than those on the observed scale. The trait PR was not heritable using either analysis method.

Live weight and height traits were moderately to highly heritable for 15-month-old heifers, 2-year-old cows and mature cows. Generally, variance components and heritabilities were larger for traits observed in mature cows compared with the same traits measured in 15-month-old heifers and 2-year-old cows and heritabilities ranged from 0.42 to 0.48 for live weight and from 0.47 to 0.65 for height traits. Adjusting MWT for BCS increased the heritability from 0.48 to 0.57. Variance components were overall lower for MWT_BCS_ compared to MWT and the largest decrease in variance was observed for the permanent environmental effect. The estimated heritability for MWT was lower following hip height adjustments (0.32). Adjustments reduced the additive genetic variance substantially but had little effect on the permanent environmental variance. Compared to MWT, the residual variance was greater for MWT_HH_. Heritability estimates for condition score traits were generally low. Similar to live weight and height traits, estimates were greater (0.26) for mature cows compared to those obtained for heifers (0.15) but did not differ from the estimates for 2-year-old females (0.25). Repeatability was high overall for mature live weight traits and ranged from 0.65 to 0.81. Estimates were also high for MHH (0.75) and moderate for BCS (0.42).

The estimated heritability was slightly greater for WWT_M_ (0.20) than for WWT_D_ (0.14). The permanent environmental effect of the dam on WWT was high (0.51). The genetic correlation for direct and maternal genetic effect of weaning weight was moderate and negative (r_am_ = −0.53).

### 3.2. Bivariate Analyses

Estimates of heritability obtained from bivariate analyses were generally similar to those from univariate analyses (Table 5). However, estimates were slightly higher for WT2 (0.52) and HH2 (0.51), as well as for MWT (0.51) and MWT_BCS_ (0.61).

The reproductive traits RB and DtC2 were highly correlated (−0.99). This is also reflected in the correlations of both reproduction traits with live weight, hip height and body condition score traits, such that the correlations with RB had the opposite sign to the correlation with DtC2. The exceptions were with WT2, HH2, BCS, MHH and WWT_D_, all of which were negatively correlated with both reproduction traits. Genetic correlations, however, were low for each of those trait combinations. Generally, 15-month-old heifer traits were moderately to highly correlated with RB (0.19–0.54) and DtC2 (−0.57–−0.23). Genetic correlations tended to decrease with increasing age and correlations changed towards the opposing sign, such that live weight, hip height and body condition traits ranged from −0.17 to −0.05 and −0.11 to 0.04 for 2-year-old cows and from −0.32 to −0.10 and −0.08 to 0.17 for mature cows for RB and DtC2, respectively. A moderate genetic correlation has been observed between WWT_M_ and RB (0.48) and the correlation with DtC2 was low and negative (−0.19).

Genetic correlations for live weight traits were lower among young animals (0.84) compared with correlations with MWT (0.94–0.96). For height traits, genetic correlations were similar among consecutive ages (0.94–0.97) and decreased with increasing age difference between traits. Estimates among condition score records were slightly lower compared with live weight and height records and ranged from 0.55 to 0.87.

Live weight and height traits were highly genetically correlated among all ages (0.61–0.85). Genetic correlations varied between live weight and condition score traits dependent on the age. Estimates were moderate among HWT and different condition scores (0.26–0.34). The highest genetic correlations were observed between WT2 and either BCS2 (0.57) or BCS (0.62) and between MWT and BCS2 (0.68) whereas no association was observed between HBCS and WT2. Body condition score traits were generally only lowly correlated with hip height measures and genetic correlations ranged from −0.16 to 0.15.

Analyses revealed high genetic correlations among WWT_D_ and all female live weight, hip height and body condition score traits among all age classes (0.51–1.00). Fifteen-month-old heifer and 2-year-old cow live weight, hip height and body condition score traits were moderately to highly genetically correlated with WWT_M_ and correlations were positive (0.32–0.74) with the exception of BCS2 (−0.40). Genetic correlations among WWT_M_ and mature cow traits were generally low to moderate and negative (−0.55–−0.22) but was low and positive between WWT_M_ and MHH (0.15).

Phenotypic correlations were lower than genetic correlations among most traits. Phenotypic correlations were overall low among female reproduction and live weight, hip height and body condition traits in 15-month-old heifers and 2-year-old cows (−0.07–0.05). Mature cow traits were lowly to moderately correlated with RB and DtC2 on a phenotypic level. Among female live weight, hip height and body condition score traits correlations were similar to genetic correlations and were generally moderate to high between live weight and height traits (0.42–0.61). Estimates among body condition score traits and live weight traits ranged from a low correlation between HBCS and MWT (0.09) to a high correlation between BCS2 and WT2 (0.57). Similar to genetic correlations, only low phenotypic correlations were observed between body condition score and height traits (−0.03–0.18). Phenotypic correlations among WWT and other live weight, hip height and body condition score traits were generally higher for 15-month-old heifers than for 2-year-old or mature cows and decreased with increasing age difference between animals.

## 4. Discussion

### 4.1. Effect of Genetics on Reproduction

It is well documented in the literature that most reproduction traits have only low heritability [24,36,37,38] and the same trend was observed in the current analyses. The estimates for HP on the underlying scale are consistent with the pooled results reported in the review by Koots et al. [18] of 0.05. McAllister et al. [39] reported slightly higher values of 0.17 in Red Angus cattle using a probit link function. On the observed scale heritabilities for yearling pregnancy rates ranged from 0.04 to 0.12 in New Zealand beef cattle [37,40]. The trait DtCH basically describes the same trait using a continuous measure of HP while also including non-pregnant females. In agreement with the low heritability estimates for HP, estimates for DtCH were equally low. Under the commercial environments the cattle were managed in as part of this study, no additive genetic variation could be detected for heifer pregnancy outcomes.

Rebreeding ability is considered a major challenge for first-calving heifers [19]. Heifers failing to conceive after their first successful calving are usually culled following weaning of that calf. Due to high costs for replacement animals and limited return from calf weaning up until this point, this is the most expensive time to replace females in the breeding herd. Reports, however, are sparse on the genetic potential for rebreeding success in 2-year-old beef cattle. Breeders are often concerned with a reduced pregnancy rate following the first successful calving [20]. This trend, however, could not be observed in the current study, such that the percentage of animals that conceived to the second mating was higher (92.0%) compared to HP (88.1%). Although heritability was low for RB on both the observed (0.14) and the underlying scale (0.12), the results obtained in this study show that genetic variation exists, such that using sires with higher genetic potential for this trait would likely result in a positive response in female progeny. Estimates published by Morris et al. [37] suggest slightly lower heritabilities for pregnancy outcomes for 2-year-old cows (0.08) on the observed scale. The high correlation between RB and DtC2 (−0.99) indicates that they are essentially measures of the same trait. Heritability for DtC2 was slightly greater (0.21) compared to RB in the current study. The greater heritability of 2-year-old cow traits indicates that breeding for greater RB and DtC2 in combination with adequate management practices has the potential to result in desirable enhancements of rebreeding ability in young herd replacements in the long term.

In the present study, PR was not heritable on either scale. This is in agreement with the low estimate of 0.04 reported by Morris et al. [37] and 0.03 by Burrow [36]. The absence of variance estimates for PR may also be attributable to animal management. Beef cows analysed for PR were subject to oestrus synchronisation prior to AI in the current study which may explain the lack of any estimable variance in the trait. Goodling et al. [41] found that oestrus synchronisation reduced the residual variance for pregnancy rate in dairy cattle but had no substantial effect on the estimated heritability in their study.

Given the different outcomes for heifers, 2-year-old females and mature cows observed in this study, the first two matings should be evaluated as different traits compared to mature cows.

### 4.2. Live Weight, Hip Height and Body Condition Score among 15-Month-Old Heifers, 2-Year-Old Cows and Mature Cows

Heritability estimates for live weight, height and body condition score traits were higher for mature cows compared to heifers and 2-year-olds and those estimates are generally in agreement with the literature. The heritability of HWT in the current study is similar to the estimate of 0.43 presented by Costa et al. [42] for US Angus heifers. Heritability estimates for mature live weight ranged from 0.29 to 0.60 dependent on breed, age, time of the year and modelling approach [18,43,44,45,46,47,48]. Mercadante et al. [15] reported lower heritabilities for heifer hip height (0.44) compared to hip height of cows (0.55) in Nellore cattle. The estimated heritability for MHH in the current study is within the range of values presented by Arango et al. [43] of 0.59–0.72 for cows between 3 and 8 years of age. The heritability of body condition score in heifers (0.09) presented by Mercadante et al. [15] was slightly lower than for mature cows (0.20) and this aligns with the range of estimates (0.16–0.21) reported by Arango et al. [43] and Johnston et al. [49] for different breeds.

Heritability estimates for height were generally greater than for live weight across all ages in the current study and this agrees with previous reports [43,46,50]. The results, however, are in contrast to estimates reported by Meyer [48] where heritabilities for hip height of cows (0.19–0.33) were generally in the same range as live weight (0.30–0.33) and were lower than the estimates reported in this study. In agreement with other studies [43,48], heritability estimates for body condition score traits were always lower than for live weight or height traits.

Adjusting MWT for BCS reduced all variance components slightly. The lower heritabilities for MWT compared to MWT_BCS_ can be primarily explained by the difference in permanent environmental variance and this agrees with the results reported by Arango et al. [43] who suggested that those differences may be attributable to variable cow environmental effects on body fat reserves that are better accounted for when adjusting MWT to a constant BCS. The higher heritability of MWT_BCS_ compared to MWT agrees with the results reported by Arango et al. [43] of 0.54 and Nephawe et al. [46] of 0.57. Meyer [48] also reported slightly higher estimates following BCS adjustments but estimates were overall lower (0.31–0.34) than in the current study (0.57).

Estimates on MWT adjusted to a constant height are rare in the literature. The results of 0.32 found in this study agree with the estimates reported by Hickson and Pitchford [51] of 0.25–0.35 in Australian Angus cows. Adjusting MWT to a standard hip height reduced the heritability considerably and this is primarily due to a reduction in the additive genetic variance. Hip height is a highly heritable trait and taking out the effect of differences in hip height may remove most of the variation in MWT that is explained through skeletal size. The majority of effects that influence MWT following hip height adjustments are likely related to muscle and fat deposition and heritability for those traits tends to be lower. Heritability for MWT_HH_ (0.32) was slightly greater than for BCS (0.26) in the current study. Genetic and phenotypic correlations among MWT_HH_ and other female live weight, hip height and body condition score traits, however, indicate that MWT_HH_ behaves in a similar manner to BCS. Hickson and Pitchford [51] found that estimating MWT_HH_ did not add significant value to BCS as a selection criterion for improved condition. In practice, height may be a more complex trait to measure compared to BCS. The advantage of this method is, however, that measuring height is a more standardised method and does not require a trained technician to accurately record body energy reserves, thus would remove bias due to subjective assessment of animals.

Repeatability estimates were high for mature cow live weights (0.65–0.81) and this is within the range of estimates reported in the literature between 0.57 and 0.85 using REML [43,47,48,49]. Burrow [36], however, reported a higher repeatability for cow live weight of 0.93. Repeatability of MHH was similar compared to live weight traits and this is in agreement with the reported estimates of 0.75 by Arango et al. [43] and 0.73 to 0.77 by Meyer [48]. Among live weight, hip height and body condition traits, BCS was the least repeatable in the current study (0.42) and the same has been observed in previous research [48]. The estimated repeatability was within the range (0.32–0.52) reported by Johnston et al. [49] for different beef cow breeds.

Genetic correlations were high among live weight and height traits and this was expected [43,48,50]. In agreement with literature findings [43], the genetic correlations were low between body condition score and height traits. Moderate to high genetic correlations were estimated in the current study between live weight and body condition score traits and this agrees with the estimates (0.49–0.65) reported by Johnston et al. [49] across different breeds. Thus, selecting for reduced mature live weight (without adjustment for BCS) to decrease maintenance requirements can reduce body condition score. Previous research has shown that lower conditioned mixed-aged cows may experience reduced reproductive performance compared to better conditioned cows on a phenotypic level [10]. Besides reproduction related reasons, other rationales, such as health and animal welfare, exist for breeders to produce cows that are able to maintain or increase BCS. The current study indicates that breeding for limited mature cow live weight and size to reduce maintenance requirements while at the same time maintaining BCS requires an alternative approach to using this information to prevent any unfavourable selection against correlated traits with potential impact on productivity.

### 4.3. Association among Reproduction, Live Weight, Hip Height and Body Condition

Results from the current study suggest a positive genetic correlation between RB and live weight, hip height and body condition score traits in 15-month-old heifers, indicating that cows with a higher genetic potential for RB performance would be likely to show increased HWT, HBCS and HH. With increasing age, the correlation decreased among RB or DtC2 and the corresponding live weight, hip height and body condition score traits toward the opposing sign. The correlations, however, were only low in 2-year-old cows (−0.17–0.04) and low to moderate in mature cows (−0.32–0.17). Given the large standard errors among those correlations with both reproductive traits, females with genetically superior reproductive performance at rebreeding are unlikely to exhibit an unfavourable reduction in body energy reserves as a cow. Comparing the genetic correlations of reproductive traits with other heifer and mature cow traits indicates that improvement in RB and DtC2 may result in faster growing, better conditioned heifers but those heifers are unlikely to become bigger cows. The correlation between DtC2 and MWT was low and positive (0.08) in the current study and was not significantly different from zero. Burrow [36] also reported a low but negative genetic correlation of −0.15 between mature live weight and days to calving. According to Mercadante et al. [15], selection for growth-related traits would not compromise reproductive performance, which in their case was measured as days to calving and calving success. Generally, results from the current analyses tend to agree with their assertion. Comparing results of DtC2 analysed in the current study with days to calving, however, needs to be treated with caution as gestation length will explain part of the variation in days to calving.

### 4.4. Maternal Contribution to Calf Weaning Weight and Its Impact on Reproduction, Live Weight, Hip Height and Body Condition Score

Heritability estimates for WWT found in the current study agree with the estimates reported by Splan et al. [52] for the direct genetic effect of WWT (0.14) and the maternal genetic effect (0.19) in US crossbred beef cattle. Similar estimates were reported by Burrow [36] in Australian Belmont cattle with 0.17 for the direct genetic and 0.34 for the maternal genetic effect of weaning weight. Those values are consistent with those reported by Morris et al. [37] of 0.14 for direct and 0.35 for maternal weaning weight. Meyer et al. [53] reported slightly higher estimates for direct weaning weight (0.22) and lower heritabilities for the maternal component of weaning weight (0.18) in Australian Hereford cattle. The permanent environmental effect of the dam was also lower in their study (0.20) compared to the result in the current analysis (0.51). Results from this study indicate that a large proportion of differences in calf weaning weights are attributable to the genetics and permanent environments of the dam after the effects of CG and age of dam have been removed.

The additive maternal genetic correlation between WWT_D_ and WWT_M_ in the current study was moderate and negative (−0.53) and an unfavourable correlation has been previously reported by several researchers [27,36,54,55,56]. Genetic correlations between the direct genetic component of WWT_D_ and live weight, hip height and body condition score traits are similar to those correlations among other live weight, height and body condition score traits and this was expected. The highest correlations were estimated between WWT_D_ and live weight and height traits of all ages which agrees with the literature [27,56,57] and estimates were generally lower among WWT_D_ and body condition score traits.

The contribution of the dams to the WWT of their calves in terms of milk production has been previously described in the literature but correlations with other maternal and production type traits are sparse. In the current study, the genetic correlations were moderate to high between WWT_M_ and live weight, hip height and body condition score traits in 15-months-old heifers. Once females calve for the first time, cows with genetics for higher WWT_M_ are likely to exhibit lower body condition scores as shown in the current study. Results from the current study are in agreement with Wolcott et al. [57] who reported a moderate negative genetic correlation between the maternal component of WWT and body condition score (−0.50) as well as low correlations with weight (0.15) and height traits (0.19) for 2-year-old Brahman cows recorded prior to their second mating season. The positive genetic correlations among WWT_M_ and any height measure observed in this study indicate that cows with greater genetic merit for height are more likely to exhibit greater overall WWT_M_ and, therefore, are likely to show greater milk production. Those correlations were, however, low at maturity and this has been previously reported for dairy cows [58]. The negative genetic correlation between MWT and WWT_M_ in the current study agrees with the estimates reported by Mwansa et al. [27] of −0.17 and Kaps et al. [54] of −0.34.

## 5. Conclusions

This study has shown that there is potential to improve reproductive performance in 2-year-old cows through selection. Animals with greater genetic potential for rebreeding performance as 2-year-olds tend to be heavier, taller heifers at 15 months of age with greater body fat reserves but those animals do not tend to be genetically bigger at maturity and this may reduce cow maintenance requirements. Traits measured on heifers prior to their first calving can be substantially different from similar traits measured in cows following their first and subsequent calving. This should be taken into account when considering maternal performance for genetic evaluations and selection program design.

## Figures and Tables

**Table 1:** Trait abbreviations, units of measurement, number (n) of records, individual animals, sires and dams, range of measurements, means and standard deviations (SD) after adjustments and scaling.

Abb.	Unit	n of Records		n of Individual Records	n of Dams	n of Sires	Range	Mean (SD)
		Females	Males					
Reproduction								
HP	%	1660	-	1660	1349	232	0/1	88.1 ^1^
DtCH	Days	1904	-	1904	1532	242	0–82	24.4 (21.3)
RB	%	1189	-	1189	1041	203	0/1	92.0 ^1^
DtC2	Days	1220	-	1220	1072	206	0–91	25.4 (21.0)
PR	%	11,730	-	4240	596	148	0/1	93.3 ^1^
Live weight, hip height and body condition								
HWT	kg	2347	-	2347	1822	328	282–444	357.0 (27.1)
HBCS	Score	2340	-	2340	1822	328	6–9	7.9 (0.6)
HH	cm	2948	-	2948	2185	358	99–133	115.3 (4.7)
WT2	kg	1488	-	1488	1265	243	299–656	470.0 (52.4)
BCS2	Score	1484	-	1484	1263	242	4–9	7.1 (0.8)
HH2	cm	1535	-	1535	1295	257	116–139	127.1 (3.9)
MWT	kg	35,375	-	4658	897	195	408–728	562.4 (47.2)
BCS	Score	35,393	-	4660	897	195	3–10	6.9 (1.0)
MHH	cm	5172	-	3552	858	186	118–143	130.3 (4.0)
WWT	kg	3454	3524	6978	3861	381	110–338	226.5 (32.1)

HP = pregnancy rate of 15-month-old heifers; DtCH = days to conception in 15-month-old heifers; RB = rebreeding performance in 2-year-old cows; DtC2 = days to conception in 2-year-old cows; PR = pregnancy rate of mature cows; HWT = live weight of 15-month-old heifers; HBCS = body condition score of 15-month-old heifers; HH = hip height of 15-month-old heifers; WT2 = live weight of 2-year-old cows; BCS2 = body condition score of 2-year-old cows; HH2 = hip height of 2-year-old cows; MWT = mature cow live weight; BCS = body condition score of mature cows; MHH = hip height of mature cows; WWT = weaning weight of calves. ^1^ Percentage.

**Table 2:** Contemporary group (CG) definitions for each trait and number (n) of CGs.

Traits	CG Definition	n of CGs
Reproduction		
HP; DtCH	Herd × recording date × birth year × birth group × weaning group × yearling group × management group at recording	59; 85
RB; DtC2	Herd × recording date × birth year × management group at recording	11; 12
PR	Herd × recording date × management group	71
Live weight, hip height and body condition		
HWT; HBCS; HH	Herd × recording date × birth group × weaning group × yearling group × management group at recording	128–137
WT2; BCS2; HH2	Herd × recording date × management group at recording	19–33
MWT; MBCS	Herd × recording date × management group	247
MHH	Herd × recording date	18
WWT	Herd × sex × recording date × birth management group × weaning management group	189

HP = pregnancy rate of 15-month-old heifers; DtCH = days to conception in 15-month-old heifers; RB = rebreeding performance in 2-year-old cows; DtC2 = days to conception in 2-year-old cows; PR = pregnancy rate of mature cows; HWT = live weight of 15-month-old heifers; HBCS = body condition score of 15-month-old heifers; HH = hip height of 15-month-old heifers; WT2 = live weight of 2-year-old cows; BCS2 = body condition score of 2-year-old cows; HH2 = hip height of 2-year-old cows; MWT = mature cow live weight; BCS = body condition score of mature cows; MHH = hip height of mature cows; WWT = weaning weight of calves.

**Table 3:** Fixed and random effects for each trait included in the variance component analyses.

	Fixed Effects					Random Effects			
	Age ^1^	Age of Dam	Breed of Animal	Heterosis	CG	Direct Genetic	Maternal Genetic	Permanent Environment	Maternal Environment
Reproduction									
HP	✓	✓	✓	✓	✓	✓			
DtCH	✓	✓	✓	✓	✓	✓			
RB	✓	✓	✓	✓	✓	✓			
DtC2	✓	✓	✓	✓	✓	✓			
PR	✓		✓	✓	✓	✓			
Live weight, hip height and body condition									
HWT	✓	✓	✓	✓	✓	✓			
HBCS	✓	✓	✓	✓	✓	✓			
HH	✓	✓	✓	✓	✓	✓			
WT2	✓	✓	✓	✓	✓	✓			
BCS2	✓	✓	✓	✓	✓	✓			
HH2	✓	✓	✓	✓	✓	✓			
MWT			✓	✓	✓	✓		✓	
MWT_BCS_			✓	✓	✓	✓		✓	
MWT_HH_			✓	✓	✓	✓		✓	
BCS			✓	✓	✓	✓		✓	
MHH			✓	✓	✓	✓		✓	
WWT		✓	✓	✓	✓	✓	✓		✓

HP = pregnancy rate of 15-month-old heifers; DtCH = days to conception in 15-month-old heifers; RB = rebreeding performance in 2-year-old cows; DtC2 = days to conception in 2-year-old cows; PR = pregnancy rate of mature cows; HWT = live weight of 15-month-old heifers; HBCS = body condition score of 15-month-old heifers; HH = hip height of 15-month-old heifers; WT2 = live weight of 2-year-old cows; BCS2 = body condition score of 2-year-old cows; HH2 = hip height of 2-year-old cows; MWT = mature cow live weight; MWTBCS = mature cow live weight adjusted for body condition score; MWTHH = mature cow live weight adjusted for hip height; BCS = body condition score of mature cows; MHH = hip height of mature cows; WWT = weaning weight of calves. ^1^ Age fitted as a linear covariate for HP, DtCH, RB, DtC2, HWT, HBCS, HH, WT2, BCS2, HH2 and factor for PR.

**Table 4:** (Co)variance components ($\sigma_{a}^{2}$ = additive genetic variance, $\sigma_{m}^{2}$ = maternal genetic variance, $\sigma_{\mathrm{am}}^{}$ = direct-maternal genetic covariance, $\sigma_{\mathrm{pe}}^{2}$ = permanent environmental variance, $\sigma_{\mathrm{me}}^{2}$ = maternal environmental variance, $\sigma_{\varepsilon }^{2}$ = residual variance), heritabilities (h^2^ ± SE) and repeatabilities (t ± SE) from univariate animal models for maternal performance traits in New Zealand beef cattle.

	**Model ^1^**	$\sigma_{a}^{2}$	$\sigma_{m}^{2}$	$\sigma_{\mathrm{am}}$	$\sigma_{\mathrm{pe}}^{2}$	$\sigma_{\mathrm{me}}^{2}$	$\sigma_{\varepsilon }^{2}$	**h^2^**	**t**
Reproduction									
HP	LM	0.00					0.11	0.00	
	THM	0.20					3.29	0.06 ± 0.08	
DtCH	LM	2.61					430.71	0.01 ± 0.05	
RB	LM	0.01					0.06	0.14 ± 0.09	
	THM	0.46					3.29	0.12 ± 0.11	
DtC2	LM	80.85					305.60	0.21 ± 0.09	
PR	LM	0.00					0.06	0.00	
	THM	0.00					3.29	0.00	
Live weight, hip height and body condition									
HWT	LM	289.19					401.08	0.42 ± 0.07	
HBCS	LM	0.02					0.14	0.15 ± 0.05	
HH	LM	5.83					5.49	0.51 ± 0.06	
WT2	LM	705.23					887.29	0.44 ± 0.09	
BCS2	LM	0.09					0.26	0.25 ± 0.08	
HH2	LM	5.57					6.16	0.47 ± 0.09	
MWT	LM	1157.14			790.86		481.64	0.48 ± 0.04	0.80 ± 0.004
MWT_BCS_	LM	952.07			403.78		324.36	0.57 ± 0.04	0.81 ± 0.004
MWT_HH_	LM	522.81			548.30		587.32	0.32 ± 0.06	0.65 ± 0.014
BCS	LM	0.15			0.10		0.34	0.26 ± 0.03	0.42 ± 0.007
MHH	LM	8.21			1.25		3.08	0.65 ± 0.05	0.75 ± 0.010
WWT_D_ ^3^	LM	84.37	122.38	−53.77		187.87	235.03	0.14 ± 0.02	
WWT_M_ ^3^	LM							0.20 ± 0.07	0.51 ± 0.03

HP = pregnancy rate of 15-month-old heifers; DtCH = days to conception in 15-month-old heifers; RB = rebreeding performance in 2-year-old cows; DtC2 = days to conception in 2-year-old cows; PR = pregnancy rate of mature cows; HWT = live weight of 15-month-old heifers; HBCS = body condition score of 15-month-old heifers; HH = hip height of 15-month-old heifers; WT2 = live weight of 2-year-old cows; BCS2 = body condition score of 2-year-old cows; HH2 = hip height of 2-year-old cows; MWT = mature cow live weight; MWTBCS = mature cow live weight adjusted for body condition score; MWTHH = mature cow live weight adjusted for hip height; BCS = body condition score of mature cows; MHH = hip height of mature cows; WWTD = direct genetic effect on calf weaning weight; WWTM = maternal genetic effect on calf weaning weight. ^1^ LM = linear model; THM = threshold model. ^3^ Only one model was fitted for WWT such that the variances presented for WWTD also apply for WWTM.

**Table 5:** Averaged heritabilities (±SEM, diagonal), genetic (±SE, below diagonal) and phenotypic (±SE, above diagonal) correlations from bivariate animal models among 15-month-old heifer, 2-year-old cow and mature cow traits in New Zealand beef cattle.

	RB	DtC2	HWT	HBCS	HH	WT2	BCS2	HH2	MWT	MWT_BCS_	MWT_HH_	BCS	MHH	WWT
RB	0.13	−0.74	0.01	0.05	−0.01	0.05	0.01	0.01	−0.25	−0.23	−0.31	−0.40	−0.20	0.02
	(0.01)	(0.01)	(0.03)	(0.03)	(0.03)	(0.03)	(0.03)	(0.03)	(0.03)	(0.04)	(0.04)	(0.03)	(0.04)	(0.04)
DtC2	−0.99	0.21	−0.03	−0.04	−0.02	−0.07	−0.06	−0.02	0.19	0.13	0.25	0.25	0.05	0.00
	(0.12)	(0.00)	(0.03)	(0.03)	(0.03)	(0.03)	(0.03)	(0.03)	(0.03)	(0.04)	(0.04)	(0.04)	(0.04)	(0.04)
HWT	0.19	−0.23	0.45	0.38	0.61	0.73	0.26	0.53	0.54	0.62	0.32	0.17	0.48	0.79
	(0.25)	(0.20)	(0.01)	(0.02)	(0.01)	(0.01)	(0.03)	(0.02)	(0.02)	(0.02)	(0.03)	(0.03)	(0.03)	(0.02)
HBCS	0.49	−0.32	0.26	0.15	0.12	0.23	0.25	0.05	0.09	0.06	0.12	0.12	−0.03	0.26
	(0.37)	(0.30)	(0.16)	(0.00)	(0.02)	(0.03)	(0.03)	(0.03)	(0.03)	(0.03)	(0.04)	(0.03)	(0.03)	(0.03)
HH	0.54	−0.57	0.71	−0.07	0.53	0.52	0.09	0.65	0.42	0.49	0.07	0.10	0.62	0.68
	(0.23)	(0.18)	(0.06)	(0.17)	(0.01)	(0.02)	(0.03)	(0.02)	(0.03)	(0.02)	(0.04)	(0.03)	(0.02)	(0.02)
WT2	−0.05	−0.11	0.84	0.00	0.66	0.52	0.57	0.54	0.74	0.79	0.47	0.35	0.59	0.57
	(0.26)	(0.21)	(0.05)	(0.20)	(0.08)	(0.03)	(0.02)	(0.02)	(0.01)	(0.01)	(0.03)	(0.03)	(0.02)	(0.03)
BCS2	−0.17	0.04	0.34	0.55	−0.13	0.57	0.27	0.09	0.33	0.24	0.33	0.40	0.18	0.17
	(0.32)	(0.27)	(0.16)	(0.23)	(0.16)	(0.12)	(0.01)	(0.03)	(0.03)	(0.03)	(0.03)	(0.03)	(0.03)	(0.03)
HH2	−0.14	−0.11	0.66	−0.03	0.94	0.61	−0.09	0.51	0.48	0.58	0.09	0.09	0.75	0.45
	(0.28)	(0.22)	(0.08)	(0.20)	(0.04)	(0.10)	(0.19)	(0.02)	(0.03)	(0.02)	(0.04)	(0.04)	(0.01)	(0.03)
MWT	−0.15	0.08	0.94	0.35	0.69	0.96	0.68	0.85	0.51	0.87	0.80	0.47	0.56	0.25 ^1^
	(0.21)	(0.10)	(0.05)	(0.12)	(0.05)	(0.03)	(0.09)	(0.05)	(0.01)	(0.00)	(0.01)	(0.01)	(0.01)	(0.02)
MWT_BCS_	−0.32	0.17	0.95	0.18	0.75	0.95	0.55	0.89	0.92	0.61	0.61	−0.01	0.62	0.31 ^1^
	(0.17)	(0.10)	(0.04)	(0.10)	(0.04)	(0.02)	(0.09)	(0.04)	(0.01)	(0.02)	(0.01)	(0.01)	(0.01)	(0.02)
MWT_HH_	−0.18	0.13	0.53	0.18	−0.04	0.69	0.50	0.16	0.71	0.60	0.31	0.56	−0.04	0.20
	(0.26)	(0.19)	(0.11)	(0.17)	(0.09)	(0.11)	(0.15)	(0.12)	(0.04)	(0.06)	(0.00)	(0.01)	(0.02)	(0.03)
BCS	−0.10	−0.08	0.26	0.61	0.01	0.62	0.87	0.11	0.24	−0.14	0.50	0.27	0.07	0.05
	(0.19)	(0.14)	(0.08)	(0.14)	(0.06)	(0.08)	(0.09)	(0.08)	(0.07)	(0.07)	(0.08)	(0.00)	(0.01)	(0.03)
MHH	−0.27	−0.04	0.61	−0.16	0.88	0.64	−0.03	0.97	0.77	0.80	0.16	0.15	0.65	0.31
	(0.18)	(0.14)	(0.07)	(0.13)	(0.05)	(0.06)	(0.12)	(0.03)	(0.04)	(0.03)	(0.09)	(0.08)	(0.00)	(0.03)
WWT_D_	−0.08	−0.05	0.87	0.52	0.56	0.70	0.51	0.69	1.00 ^1^	0.99 ^1^	0.64	0.89	0.53	0.16
	(0.28)	(0.23)	(0.05)	(0.16)	(0.08)	(0.10)	(0.17)	(0.11)	(0.07)	(0.06)	(0.18)	(0.12)	(0.14)	(0.01)
WWT_M_	0.48	−0.19	0.71	0.32	0.74	0.39	−0.40	0.33	−0.28 ^1^	−0.22 ^1^	−0.36	−0.55	0.15	−0.53 ^2^
	(0.28)	(0.20)	(0.14)	(0.18)	(0.12)	(0.14)	(0.19)	(0.14)	(0.09)	(0.08)	(0.12)	(0.12)	(0.09)	(0.17)

RB = rebreeding performance in 2-year-old cows; DtC2 = days to conception in 2-year-old cows; HWT = live weight of 15-month-old heifers; HBCS = body condition score of 15-month-old heifers; HH = hip height of 15-month-old heifers; WT2 = live weight of 2-year-old cows; BCS2 = body condition score of 2-year-old cows; HH2 = hip height of 2-year-old cows; MWT = mature cow live weight; MWTBCS = mature cow live weight adjusted for body condition score; MWTHH = mature cow live weight adjusted for hip height; BCS = body condition score of mature cows; MHH = hip height of mature cows; WWTD = direct genetic effect on calf weaning weight; WWTM = maternal genetic effect on calf weaning weight. ^1^ Convergence only achieved if parameters were allowed to exceed limits of the parameter space (i.e., correlations >1.00 for direct genetic correlations). ^2^ Genetic correlation between WWTM and WWTD. The underline has been added to highlight the values on the diagonal (heritabilities).

## Data Availability

Third-Party Data. Restrictions apply to the availability of these data. Data were obtained from Beef + Lamb New Zealand Genetics.
